# Unexpected High Losses of *Anopheles gambiae* Larvae Due to Rainfall

**DOI:** 10.1371/journal.pone.0001146

**Published:** 2007-11-07

**Authors:** Krijn P. Paaijmans, Moses O. Wandago, Andrew K. Githeko, Willem Takken

**Affiliations:** 1 Meteorology and Air Quality, Wageningen University, Wageningen, The Netherlands; 2 Laboratory of Entomology, Wageningen University, Wageningen, The Netherlands; 3 Climate and Human Health Research Unit, Kenya Medical Research Institute, Kisumu, Kenya; University of Sydney, Australia

## Abstract

**Background:**

Immature stages of the malaria mosquito *Anopheles gambiae* experience high mortality, but its cause is poorly understood. Here we study the impact of rainfall, one of the abiotic factors to which the immatures are frequently exposed, on their mortality.

**Methodology/Principal Findings:**

We show that rainfall significantly affected larval mosquitoes by flushing them out of their aquatic habitat and killing them. Outdoor experiments under natural conditions in Kenya revealed that the additional nightly loss of larvae caused by rainfall was on average 17.5% for the youngest (L1) larvae and 4.8% for the oldest (L4) larvae; an additional 10.5% (increase from 0.9 to 11.4%) of the L1 larvae and 3.3% (from 0.1 to 3.4%) of the L4 larvae were flushed away and larval mortality increased by 6.9% (from 4.6 to 11.5%) and 1.5% (from 4.1 to 5.6%) for L1 and L4 larvae, respectively, compared to nights without rain. On rainy nights, 1.3% and 0.7% of L1 and L4 larvae, respectively, were lost due to ejection from the breeding site.

**Conclusions/Significance:**

This study demonstrates that immature populations of malaria mosquitoes suffer high losses during rainfall events. As these populations are likely to experience several rain showers during their lifespan, rainfall will have a profound effect on the productivity of mosquito breeding sites and, as a result, on the transmission of malaria. These findings are discussed in the light of malaria risk and changing rainfall patterns in response to climate change.

## Introduction

With over a million deaths and between 350 and 500 million acute cases annually [1], malaria remains one of the most important and widespread tropical infectious diseases in the world. Over 75% of the fatal cases occur among children living in sub-Saharan Africa [2]. In this region, two sibling mosquito species *Anopheles arabiensis* Patton and *An. gambiae* Giles sensu stricto (hereafter referred to as *An. gambiae*) both belonging to the *An. gambiae* sensu lato complex and *An. funestus* Giles, are the principle vectors of malaria.

The immature stages of *An. gambiae* require an aquatic environment to develop and are often found in transient, sunlit and generally small pools [3]–[6]. The availability of these aquatic habitats depends on precipitation [6]–[8]. Precipitation creates new breeding sites and adds water to existing ones. The availability, persistence and dimensions of mosquito larval habitats depend to a large extent on the frequency, duration and intensity of precipitation.

Mortality during the development of the larval stages is very high. Various studies have reported that only a small fraction (2–8%) of the larvae that hatched eventually survived to the adult stage and attributed this to the presence or absence of predators, parasites, pathogens [9]–[12] or cannibalism [13]. Other biotic factors that may affect survival are predation by sibling species [14] and other interactions between sibling species [15]. Abiotic factors such as temperature [16]–[18] may also affect larval mortality.

It has been suggested that precipitation could affect larval population dynamics by flooding habitats and consequently flushing out larvae [19]–[22]. Tuno et al. [23] observed a high larval mortality in open habitats in the western Kenya highlands and suggested a damaging effect of raindrops on larvae. The possible effect of mortality by the direct hit of a raindrop was studied by Mason [in: 19], who exposed larvae to rain showers and by Robert et al. [24], who exposed larvae to artificial rain. However, in both studies no damaging effect was observed. Russell et al. [19] proposed that the direct damage to anopheline larvae by precipitation may depend on raindrop size.

The Intergovernmental Panel on Climate Change (IPCC) expects significant variation in rainfall in tropical Africa in response to global warming, whereby East Africa in particular is likely to experience an increase in annual mean rainfall [25]. Hulme et al. [26] predicted a similar pattern in equatorial East Africa but the expected increase in rainfall during December-February varied from 5–30% to 50–100% depending on the speed of global warming. An increase in rainfall will increase the availability, persistence and dimensions of larval habitats, although this will depend on parameters such as local evaporation rates, soil percolation and slope of the terrain [27]. Moreover, an increase in rainfall may have negative consequences for mosquito populations by impacting the immature life stages through excessive flooding or by direct hits.

The biotic and abiotic factors that affect life history traits such as growth, development and survival of the immature stages of *An. gambiae* s.l. require more attention, as they will affect productivity in the breeding site and determine the abundance, distribution and fitness of the resultant adult mosquito populations, which will consequently affect the malaria transmission. Here we explore the effect of natural rainfall, a density-independent factor, on flushing, ejection and mortality of larvae of the malaria vector *An. gambiae* under ambient conditions in western Kenya.

## Results

### Experiment I-Flushing and Mortality of *An. gambiae* Larvae

#### Meteorological Data

**Figure 1:**
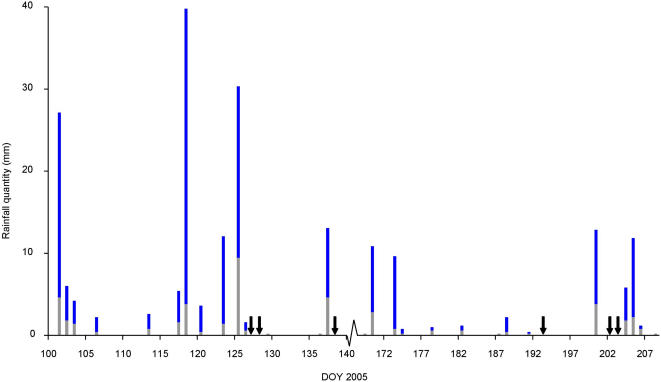
Rainfall during the study period. Total rainfall quantity, including maximum rainfall intensity (in grey), per night from April 10 (Day Of Year 100) up to July 27 (DOY 208). The arrows indicate missing data. Note that DOY 141 up to 167 are omitted from the figure, as no experiments were carried out.

Experiments were carried out on 45 nights without and on 26 nights with rainfall. The total quantity of rainfall varied from 0.2 to 39.8 mm per night and the maximum rainfall intensity recorded was 9.5 mm in 5 minutes. During nights with rainfall, the total rainfall quantity was significantly correlated with the highest rainfall intensity (ρ = 0.95, p<0.001) and with the duration of precipitation (ρ = 0.93, p<0.001). Highest precipitation intensity and precipitation duration were also significantly correlated (ρ = 0.79, p<0.001). Figure 1 shows the total quantity of rainfall and the maximum rainfall intensity (per 5 minutes) per night during the study period.

Comparing nights with and without rainfall, there was no significant difference in the average air temperature and the average wind speed. The average air temperature was 21.6 (S.E.M.±0.1, range 18.8–24.2)°C and 20.8 (±0.1, range 18.5–24.1)°C and the average wind speed 0.7 (±0.0, range 0.4–1.0) m/s and 0.7 (±0.0, range 0.5–1.0) m/s on nights without and with rainfall, respectively.

The average maximum recorded wind speed was significantly (p<0.05) higher during nights with rainfall (3.4±0.3, range 1.6–6.3 m/s) than during nights without rainfall (2.7±0.3, range 1.0–10.9 m/s).

#### Flushing

**Figure 2:**
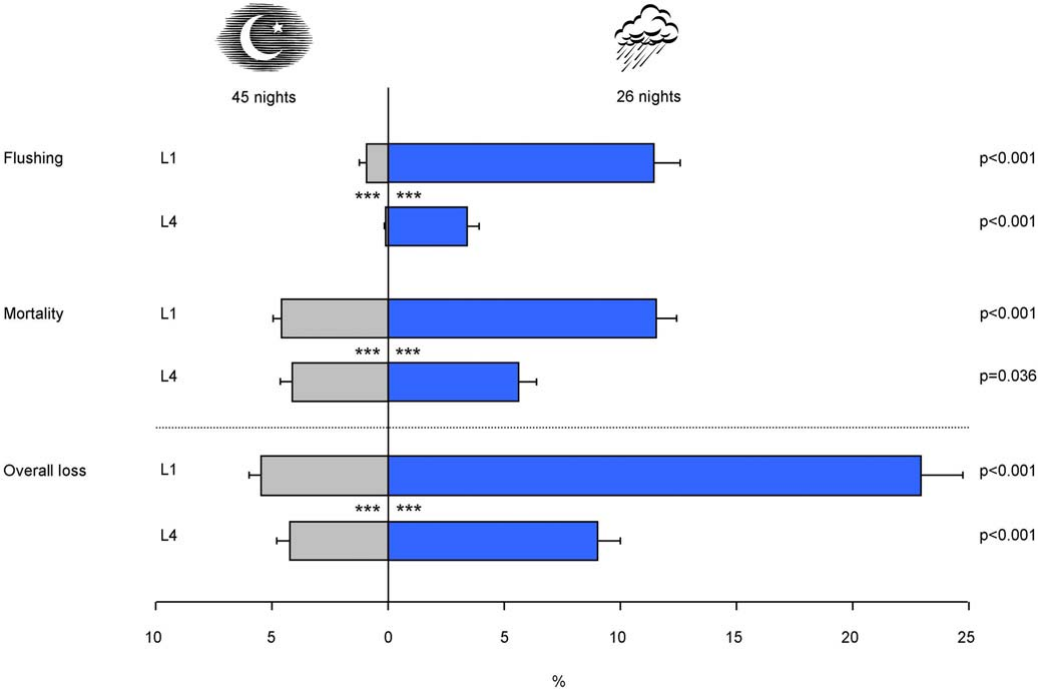
Losses of *Anopheles gambiae* larvae during nights with and without rainfall. Percentages of L1 and L4 larvae of *An. gambiae* that were flushed away or died and the overall loss during nights without rainfall (on the left) and nights with rainfall (on the right). The asterisks (***) indicate the level of significance between L1 and L4 larvae (p<0.001). The error bars indicate the standard error of the mean.

Although this was unexpected, some larvae (on average 0.9±0.3% and 0.1±0.1% of the first instar (L1) and fourth instar (L4) larvae, respectively) were swept out of the basins by flushing during nights without rainfall (Figure 2). There was no significant difference between the percentages of larvae (for both L1 and L4 larvae) that flushed out of the small and out of the large basins. L1 larvae had a higher chance (p<0.001) of being flushed than L4 during a night without rainfall (Figure 2). Flushing of L1 larvae during nights without rainfall was significantly correlated with average wind speed (ρ = 0.13, p<0.05), but not significantly correlated with highest wind speed recorded that night. Flushing of L4 larvae was not significantly correlated with either of those variables.

During nights that experienced rainfall, there was no significant difference between the percentages of larvae (for both L1 and L4 larvae) that flushed out of the small and out of the large basins. Significantly (p<0.001) more L1 and L4 larvae were swept away, during nights with rainfall compared to nights without rainfall (Figure 2). The increase in flushing was 10.5% (increase to 11.4±1.2%) for L1 larvae and 3.3% (increase to 3.4±0.5%) for L4 larvae. During nights with rainfall, L1 larvae had a significantly (p<0.001) higher chance of being flushed out than L4 larvae.

#### Mortality

Mortality of larvae after a night with rainfall was considerably higher than after a night without rainfall for both first and fourth larval stages (Figure 2). The mean increase in mortality during rainy nights was 6.9% (from 4.6±0.3 to 11.5±0.9%) and 1.5% (from 4.1±0.6 to 5.6±0.8%) for L1 and L4 larvae, respectively. On all nights, with or without rain, the survival of L1 larvae was significantly (p<0.001) lower than that of L4 larvae (Figure 2).

#### Overall Loss

Adding the average percentage of mosquito larvae that flushed out of their habitat to the average percentage of larvae that did not survive an experimental night gives the average total loss of the immature mosquito population per night. This amounted to a loss of 23% for L1 larvae and 9% for L4 larvae during a night with rainfall. During nights without rainfall these losses were 5.5% and 4.2% for L1 and L4 larvae, respectively, caused mostly by natural mortality. After correcting for the latter, the average increase in overall loss was 17.5% for L1 and 4.8% for L4 larvae during rainfall events.

### Experiment II-Ejection of *An. gambiae* Larvae

#### Meteorological Data

Experiments were carried out during 29 nights without and 16 nights with rainfall. The total quantity of precipitation varied from 0.2 to 30.4 mm per night. The maximum rainfall intensity recorded was 9.5 mm in 5 minutes. Comparing nights with and without rainfall, there was again no significant difference in the average air temperature and the average wind speed.

#### Ejection

No mosquito larvae were ejected from their original basin during nights without rainfall. During nights with rainfall, 1.3 (±0.6)% of the L1 larvae and 0.7 (±0.4)% of the L4 larvae were ejected from their original basin. The difference between nights without and with rainfall was significant for L1 (p = 0.001) and L4 (p<0.05) larvae but the chance of being ejected was similar for L1 and L4 larvae. When ejection occurred, more larvae were recovered from the medium tray (short distance) than from the large tray (long distance). There was no significant difference between the total percentage of larvae being ejected and the percentage of larvae being ejected into the medium basin.

## Discussion

Precipitation flushed, ejected and killed a significant proportion of larvae of *An. gambiae* in different stages of development. Young larvae (L1 stage) experienced the highest flushing, ejection and mortality, while the oldest larvae (L4 stage) were better able to withstand the effects of precipitation. We did not investigate the impact of rainfall on the second and third instar larval stages, but assume that their respective loss values lie within those found for the L1 and L4 stages.

The observed flushing of larvae on nights without rainfall was significantly correlated with wind speed. We occasionally observed that the water rippled due to gusts of wind and drops of water washed over the rim of the basins. Because larvae tended to be situated at the air-water interface at the rim of the basins (personal observations) and it is known that they aggregate [7], [9], larvae may have been flushed out of the basin by the turbulence caused by wind.

Under natural circumstances, breeding sites must fill up gradually before water runoff takes place. Most of the larval flushing will be related to runoff that creates small temporary streams, rather than rain that falls directly into the habitat. This is clearly related to the hydrology and shape of the larval habitats and cannot be easily replicated in an experiment. The basins in the present study were filled to the brim, which may have resulted in a larger number of larvae being flushed out during rainfall compared to sites that need to fill up first. On the other hand the basins were levelled, which may have led to an underestimation of the proportion of larvae that were flushed out. Under natural circumstances, water runoff will be stronger, as this will be concentrated at the lowest point of the edge of a breeding site instead of all around the edge as was the case in the experimental setup.

It appeared difficult to recover dead larvae because of the soil particles that had accumulated in the basins due to splashing around the basins. Therefore, larvae that were unaccounted for could either have died in the setup or have been ejected from the experimental basins. Theoretically, larvae that were categorized as flushed out could also have been ejected into the overflow basins. Ejection may have occurred if larvae were present at the air-water interface, just outside the centre of a raindrop-impact. When a raindrop hits the water surface, water is splashed away. It is conceivable that these spatters could contain a mosquito larva and that this larva is then flung away. However, our second experiment showed that the effects of ejection of larvae from the breeding site by precipitation were small, although significant. Ejection cannot occur on dry nights, so any loss caused by ejection on rainy nights will be noted but is unlikely to add greatly to larval flushing and mortality. This is similar to an earlier finding of Robert et al. [24] who observed a weak dispersal by ejection of larvae of *An. arabiensis* when they were exposed to artificial rain.

Being flushed out of or ejected from their habitat onto the muddy surroundings does not necessary imply the death of mosquito larvae. Larvae of *An. gambiae* s.l. are able to move actively over moist soil [28], [29] and may therefore reach a new body of water or return to the same one. Moreover, larvae may flow passively with runoff over the soil [29]. The percentage of immatures that reaches a new habitat remains unknown and will depend on a variety of factors, such as the duration and quantity of rainfall, geographical parameters and distance to nearest body of water. However, larvae that are swept out of their natural habitat are likely to suffer higher mortalities then those that are left in the breeding site.

The observed higher mortality of mosquito larvae after rainfall may have several causes. Serious damage may be inflicted by the force of the impact of the drop when a larva is present near the water surface in the centre of a raindrop-impact. A small experiment whereby *An. arabiensis* was exposed to artificial rain showed no mortality as a result of the shock due to raindrops [24]. Russell [19] mentioned that the direct damage done to anophelines by rain will conceivably depend on size of the raindrops.

Another explanation for the observed mortality is the occurrence of water currents during rainfall. A longer period of turbulent water may exhaust mosquito larvae if they actively try to move away from the water surface to avoid being hit by a raindrop or to avoid being flushed out of their habitat, or if they actively try to reach the air-water interface for oxygen. It was observed that larvae were less present at the air-water interface during rainfall when they were not shielded from rain (KPP, unpublished data) and it is known that the diving behaviour of larvae of *An. gambiae* can kill them [30], as such behaviour has energetic costs. When larvae are not able to obtain oxygen at the air-water interface, they can survive from 8.5 to 10.6 hours, depending on the larval stage [31], but this has not been tested in turbulent waters.

The significant loss of larvae due to rainfall will as a result decrease the larval density in a breeding site, which will lead to a lower competitive pressure for food and space. Whether such lower densities are advantageous for the development time and survival of the immatures of *An. gambiae* is not clear, as various studies in which the effect of density on mosquito life-history traits was examined, are ambiguous. In two laboratory studies, survivorship decreased at higher densities [15], [16], although the latter study showed that density strongly interacted with rearing temperature. In contrast, another laboratory study [32] showed that increasing densities (up to 2.6 larvae/cm^2^) had no effect on survivorship and similar results were obtained when larvae were reared outdoors in artificial habitats in Kenya [33]. In the same outdoor experiment, the development time was reduced when larval densities were lower [33]. One laboratory study showed a similar result [32], whereas in another study the age at pupation under laboratory conditions was shorter when densities increased [16]. The observed differences are probably due to differing set-ups of the studies and therefore more research is required to examine the relationship between the density and the development and survival of the immatures.

Rainfall may also affect the mosquito larvae indirectly, by flushing out the predators and pathogens that may have previously colonized the same habitats. This could increase the survival of mosquito larvae. Furthermore, rainfall decreases the water temperature [34], as the raindrop temperature is less than that of surface water, and this decrease, which is larger in smaller water pools (KPP, unpublished data), may affect larval development and survival as well.

The effects of rainfall on other malaria mosquito species, e.g., *An. arabiensis* and *An. funestus* may be different and need to be studied in more detail. A recent study [31] showed that both *An. arabiensis* and *An. gambiae* express a similar diving behaviour, but this was different than that of *An. funestus*, which dives less frequently. Charlwood & Edoh [35] suggested a difference in rainfall tolerance between larvae of *An. gambiae* and *An. arabiensis*, based on their numbers prior to and after heavy rainfall.

Besides investigating other malaria mosquito species, the effects of rainfall on the pupal stage of *An. gambiae* needs to be examined. This life stage is very important, as the emergence of adult mosquitoes determines the productivity of a breeding site. Romoser and Lucas [36] suggested that pupal diving behaviour during heavy pelting rains helps them to avoid drowning and being flushed away. Pupae have a ventral air space containing gas, which may be disrupted by a direct hit from a raindrop, causing the loss of hydrostatic balance. Once this balance and buoyancy are affected, they cannot be restored and the pupae eventually drown [37].

Furthermore, the long-term effects of precipitation on *An. gambiae* immatures require more attention. Rainfall may result in larval stress and larvae may have to consume more energy during rainfall, which may affect life history traits such as development time, survival and adult size.

This study showed that rainfall killed *An. gambiae* larvae and flushed them out of their habitat, resulting in additional nightly losses of 17.5% of L1 larvae and 4.8% of L4 larvae, compared to nights without rain. Mortalities of the second and third instar larvae are likely to lie in between these values. Our data showed the loss experienced by rainfall during one night only. It is likely that larvae will be exposed to more frequent rain showers during their lifespan, resulting in a large population decrease due to flushing and mortality. During our study it rained on average once every three nights, although there were periods with daily rainfall and periods of several days without rainfall. Combined with the knowledge that larvae of *An. gambiae* s.l. may take between one and three weeks to develop into adult mosquitoes under ambient conditions in the field [22], [33], larvae may experience three or more nights with rainfall during their lifespan. The proportion of larvae of one generation that may be flushed away and killed during their lifespan will be substantial and therefore rainfall per se will affect larval population dynamics dramatically.

These effects of precipitation on mosquito populations should be considered in the light of climate change and malaria risk. Small changes in temperature and precipitation would directly affect the development of parasites and the behaviour and geographical distribution of the vectors [38], [39]. If the predicted increases in rainfall in East Africa [25], [26] were to occur, the spacing of the rains will be an important determinant affecting the rates at with which mosquito populations will grow and efficiency with which they will transmit diseases [40]. Increases in rainfall will directly result in an increase in the number and a longer persistence of aquatic habitats. However, such increases will result in higher numbers of larvae that are flushed away and a higher larval mortality, as we observed moderate but significant correlations between larval flushing, ejection and mortality (especially of L1 larvae; Table 1) and rainfall quantity, maximum intensity and duration. Therefore, more rainfall not only results in more mosquito breeding sites, which may in turn lead to an increase in malaria vectors, but it will also result in a large decrease of the existing immature populations, which leads to a reduction in emerging adults of that generation. Therefore, spacing of rainfall events should be viewed as an important determinant in the productivity of *An. gambiae* breeding sites, and hence in the mosquito population dynamics and transmission of malaria. Climate change, causing increased precipitation and frequency of rain showers, may thus indirectly affect malaria and other mosquito-borne diseases by impacting the larval populations.

**Table 1:** Correlation between flushing, mortality and ejection of L1 and L4 instar larvae of *An. gambiae* and various rainfall variables during nights with rainfall.

	Flushing	Mortality	Ejection
**L1 larvae**			
Rainfall quantity (mm night^−1^)	0.49***	0.25***	0.49***
Rainfall max. intensity (mm 5 min^−1^)	0.36***	0.18*	0.47**
Rainfall duration (minutes night^−1^)	0.57***	0.28***	0.36*
**L4 larvae**			
Rainfall quantity (mm night^−1^)	0.30***	ns	0.39**
Rainfall max. intensity (mm 5 min^−1^)	0.23***	ns	0.35*
Rainfall duration (minutes night^−1^)	0.38***	ns	0.33*

Spearman's rank correlation coefficients and their level of significance are given.

(*) p<0.05

(**) p<0.01

(***) p<0.001

(ns) not significant

## Materials and Methods

### *Anopheles gambiae* Mosquitoes

Outdoor experiments were carried out on the grounds of the Kenya Medical Research Institute (KEMRI) in Kisumu, Kenya and started at 17:00 h (or one hour earlier at times when a rain shower was developing). Newly hatched L1 or L4 larvae of *An. gambiae* (Kisumu strain, maintained in the Vector Biology Control and Research Center at KEMRI) were used and they were fed 0.3 mg Tetramin®*Baby* fish food (TetraWerke, Melle, Germany) per larva at the start of each experiment. The tap water used in the experiments originated from a well at KEMRI and was stored in large containers for a few days prior to the experiments, to allow the sediments and other inorganic particles to settle. The next morning (09:00 h) all experimental basins were examined twice for larvae and their numbers (*dead*, *alive* and *not recovered*) recorded.

### Experiment I-Flushing and Mortality of *An. gambiae* Larvae

**Figure 3:**
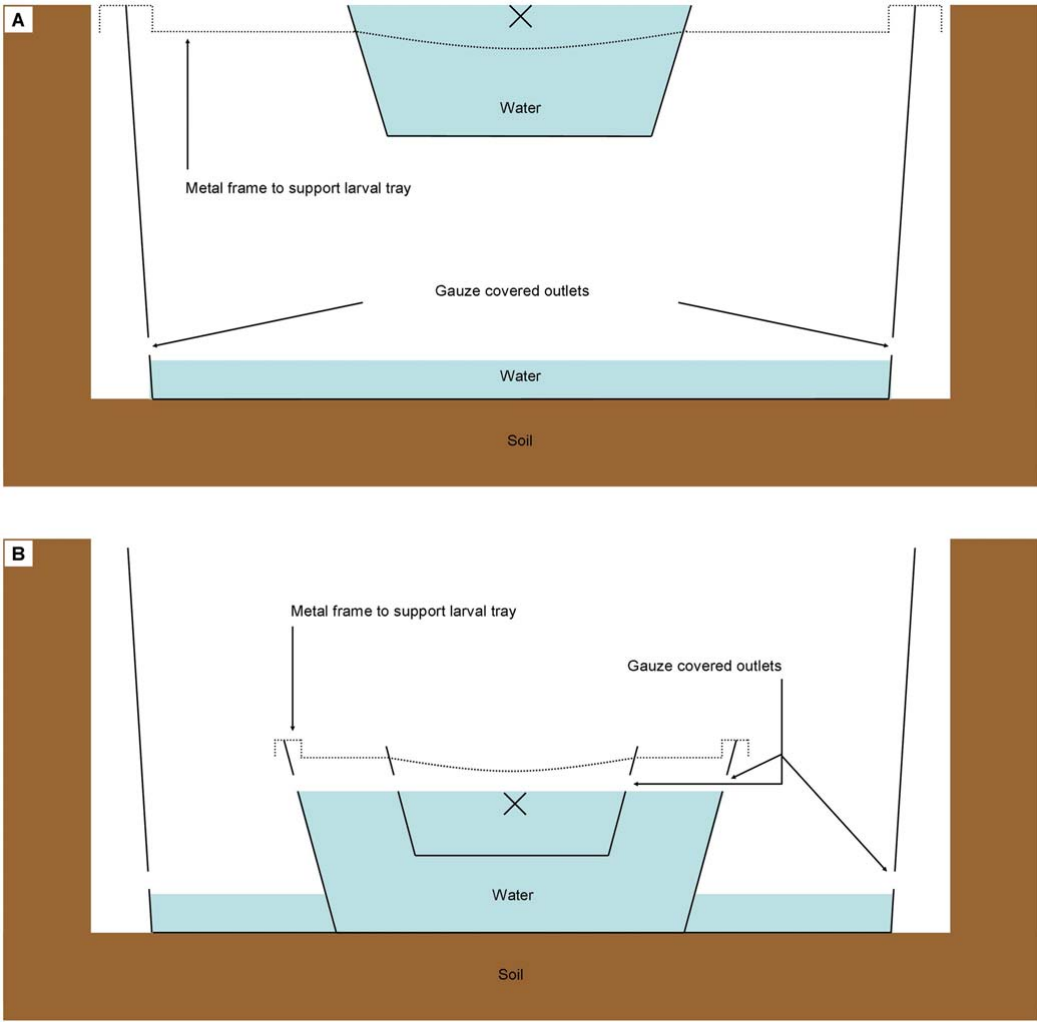
Experimental setup. (A), Schematic side view of flush/survival experiment. (B), Schematic side view of ejecting-experiment. The solid lines represent the basins; the dotted lines the thin tripod metal frames. The openings in the basins are the overflow holes screened with mesh-wire; the black crosses indicate where the larvae were placed at the start of each experiment.

To asses the extent of flushing of the larvae during rainfall and to see whether rainfall is a noticeable mortality factor, an experiment was carried out from April to July 2005, a period that covered part of the long rainy season that occurs annually in western Kenya. Small-sized (Ø 16 cm, 5 cm deep; hereafter referred to as *small basin*) or large-sized (Ø 30 cm, 9.5 cm deep; *large basin*) circular plastic basins were placed separately in the middle of a larger basin (Ø 41 cm, 16 cm deep; *overflow basin*). By using a thin metal frame, the rims of the small/large basin and the overflow basin were placed at the same height. The experimental setup (Figure 3A) was levelled horizontally and the small and large basins were filled with water to overflowing. Each overflow basin was filled with 2 cm water, to prevent larvae from desiccating after flushing, and was provided with two screened (0.20 mm mesh size) outlets (Ø 1 cm in diameter) to allow excess rainwater to run off but prevent larvae from flushing out of the overflow basin during precipitation. The overflow basins were placed a few centimetres apart in a trench so that the upper edge of the experimental setup was at the same level as its surrounding soil and excess rainwater could run off freely. At the start of each experiment (17:00 h), twenty L1 or L4 larvae were placed in the small and large basins, each immature stage having four replicates in each size basin.

### Experiment II-Ejection of *An. gambiae* Larvae

Because ejection of larvae from the basins caused by the impact of raindrops was suspected, we subsequently studied the possible occurrence of this phenomenon in a separate experiment from May to July 2005. A plastic basin (Ø 16 cm, 5 cm deep; hereafter referred to as *small basin*) was placed inside a larger plastic basin (Ø 30 cm, 9.5 cm deep; *medium sized basin*) and a thin metal frame kept the rims of the basins at the same level. The medium sized basin was placed inside a larger plastic basin (Ø 54.5 cm, 19 cm deep; *large basin*). For reasons mentioned in experiment 1, each basin was provided with two screened (0.20 mm mesh size) outlets (Ø 1 cm in diameter) and the large basins were placed a few centimetres from each other in a trench. The experimental setup (Figure 3B) was filled with water until the water level in all basins had reached the overflow outlets. At the start of each experiment (17:00 h), twenty L1 or L4 larvae were placed in the small basin, each immature stage having three replicates.

### Meteorological Data

The quantity of rainfall (mm) was measured with an automated rain gauge (Eijkelkamp, The Netherlands; opening at 0.9 meters height; threshold 0.201 mm), wind speed (m/s) was recorded two meters above ground with a cup anemometer (Meteorology and Air Quality, Wageningen University, The Netherlands) and the air temperature (°C) was measured two meters above ground with a shielded and ventilated probe (Vaisala, Finland). Every 5 minutes, total quantity of precipitation and average wind speed and air temperature were stored on a data logger (21× MicroDataLogger, Campbell Scientific, U.K.).

### Statistical Analysis

*Flushing* was calculated as the percentage of living larvae found in the overflow basin out of the total number of larvae that survived and *mortality* as the percentage of dead larvae in all basins out of the total number of larvae at the start of the experiment. *Ejection* was calculated as the percentage of living larvae recovered outside the small basin out of the total number of larvae that survived. Larvae that were not recovered were assumed dead. Larvae that had died were excluded from the *flushing* and *ejection* analysis as they were unable to respond actively to the rainfall. An L4 larva occasionally moulted to a pupa. These pupae were excluded from the analysis. Data were analyzed with the SPSS software (v. 14.0, SPSS Inc., Chicago, IL), using the Mann-Whitney *U* test. Correlations between *flushing*, *mortality* and *ejection* on the one hand and rainfall and other weather variables during an experimental night on the other hand were obtained with the Spearman's rank correlation test.

## References

[1] WHO 2005 World Malaria Report 2005. Geneva World Health Organization293Report number WHO/HTM/MAL/2005.1102.

[2] Breman JG (2001). The ears of the hippopotamus: manifestations, determinants, and estimates of the malaria burden.. American Journal of Tropical Medicine and Hygiene.

[3] Gillies MT, Coetzee M (1987). A supplement to the anophelinae of Africa south of the Sahara (Afrotropical region). Publication no. 55..

[4] Gillies MT, DeMeillon B (1968). The anophelinae of Africa south of the Sahara (Ethiopian zoogeographical region). Publication no. 54..

[5] Service MW (1993). Mosquito ecology. Field sampling methods..

[6] Gimnig JE, Ombok M, Kamau L, Hawley WA (2001). Characteristics of larval anopheline (Diptera: Culicidae) habitats in western Kenya.. Journal of Medical Entomology.

[7] Koenraadt CJM, Githeko AK, Takken W (2004). The effects of rainfall and evapotranspiration on the temporal dynamics of *Anopheles gambiae* s.s. and *Anopheles arabiensis* in a Kenyan village.. Acta Tropica.

[8] Fillinger U, Sonye G, Killeen G, Knols BGJ, Becker N (2004). The practical importance of permanent and semipermanent habitats for controlling aquatic stages of *Anopheles gambiae sensu lato* mosquitoes: operational observations from a rural town in western Kenya.. Tropical Medicine and International Health.

[9] Service MW (1971). Studies on sampling larval populations of the *Anopheles gambiae* complex.. Bulletin of the World Health Organization.

[10] Service MW (1973). Mortalities of the larvae of the *Anopheles gambiae* Giles complex and detection of predators by the precipitin test.. Bulletin of Entomological Research.

[11] Service MW (1977). Mortalities of the immature stages of species B of the *Anopheles gambiae* complex in Kenya: comparison between rice fields and temporary pools, identification of predators, and effects of insecticidal spraying.. Journal of Medical Entomology.

[12] Aniedu I, Mutinga MJ, Mutero CM (1993). Vertical estimates of survivorship of larvae and pupae of *Anopheles gambiae* Giles complex in Baringo district, Kenya.. Insect Science and its Application.

[13] Okogun GRA (2005). Life-table analysis of *Anopheles* malaria vectors: generational mortality as tool in mosquito vector abundance and control studies.. Journal of Vector Borne Diseases.

[14] Koenraadt CJM, Takken W (2003). Cannibalism and predation among larvae of the *Anopheles gambiae* complex.. Medical and Veterinary Entomology.

[15] Schneider P, Takken W, McCall PJ (2000). Interspecific competition between sibling species larvae of *Anopheles arabiensis* and *An. gambiae*.. Medical and Veterinary Entomology.

[16] Lyimo EO, Takken W, Koella JC (1992). Effect of rearing temperature and larval density on larval survival, age at pupation and adult size of *Anopheles gambiae*.. Entomologia Experimentalis et Applicata.

[17] Bayoh MN, Lindsay SW (2004). Temperature-related duration of aquatic stages of the Afrotropical malaria vector mosquito *Anopheles gambiae* in the laboratory.. Medical and Veterinary Entomology.

[18] Bayoh MN, Lindsay SW (2003). Effect of temperature on the development of the aquatic stages of *Anopheles gambiae* sensu stricto (Diptera: Culicidae).. Bulletin of Entomological Research.

[19] Russell PF, West LS, Manwell RD (1946). Practical Malariology..

[20] Mutuku FM, Bayoh MN, Gimnig JE, Vulule JM, Kamau L (2006). Pupal habitat productivity of *Anopheles gambiae* complex mosquitoes in a rural village in western Kenya.. American Journal of Tropical Medicine and Hygiene.

[21] Edillo FE, Touré YT, Lanzaro GC, Dolo G, Taylor CE (2004). Survivorship and distribution of immature *Anopheles gambiae* s.l. (Diptera: Culicidae) in Banambani village, Mali.. Journal of Medical Entomology.

[22] Jepson WF, Moutia A, Courtois C (1947). The malaria problem in Mauritius: The bionomics of Mauritian anophelines.. Bulletin of Entomological Research.

[23] Tuno N, Okeka W, Minakawa N, Takagi M, Yan G (2005). Survivorship of *Anopheles gambiae* sensu stricto (Diptera: Culicidae) larvae in western Kenya highland forest.. Journal of Medical Entomology.

[24] Robert V, Planchon O, Lapetite J-M, Esteves M (1999). Rainfall is not a direct mortality factor for anopheline larvae.. Parasite.

[25] Christensen JH, Hewitson B, Busuioc A, Chen A, Gao X, Solomon S, Qin D, Manning M, Chen Z, Marquis M (2007). Regional climate projections.. Climate change 2007: the physical science basis, contribution of working group I to the fourth assessment report of the Intergovernmental Panel on Climate Change.

[26] Hulme M, Doherty R, Ngara T, New M, Lister D (2001). African climate change: 1900–2100.. Climate Research.

[27] McMichael AJ, Haines A, Slooff R, Kovats S (1996). Climate change and human health: an assessment prepared by a task group on behalf of the World Health Organization, the World Meteorological Organization and the United Nations Environment Programme..

[28] Koenraadt CJM, Paaijmans KP, Githeko AK, Knols BGJ, Takken W (2003). Egg hatching, larval movement and larval survival of the malaria vector *Anopheles gambiae* in desiccating habitats.. Malaria Journal.

[29] Miller JR, Huang J, Vulule J, Walker ED (2007). Life on the edge: African malaria mosquito (*Anopheles gambiae* s.l.) larvae are amphibious.. Naturwissenschaften.

[30] Tuno N, Miki K, Minakawa N, Githeko A, Yan G (2004). Diving ability of *Anopheles gambiae* (Diptera: Culicidae) larvae.. Journal of Medical Entomology.

[31] Tuno N, Githeko A, Yan G, Takagi M (2007). Interspecific variation in diving activity among *Anopheles gambiae* Giles, *An. arabiensis* Patton, and *An. funestus* Giles (Diptera: Culicidae) larvae.. Journal of Vector Ecology.

[32] Timmermann SE, Briegel H (1993). Water depth and larval density affect development and accumulation of reserves in laboratory populations of mosquitoes.. Bulletin of the Society for Vector Ecology.

[33] Gimnig JE, Ombok M, Otieno S, Kaufman MG, Vulule JM (2002). Density-dependent development of *Anopheles gambiae* (Diptera: Culicidae) larvae in artificial habitats.. Journal of Medical Entomology.

[34] Bayoh MN (2001). Studies on the development and survival of *Anopheles gambiae* sensu stricto at various temperatures and relative humidities..

[35] Charlwood JD, Edoh D (1996). Polymerase chain reaction used to describe larval habitat use by *Anopheles gambiae* complex (Diptera: Culicidae) in the environs of Ifakara, Tanzania.. Journal of Medical Entomology.

[36] Romoser WS, Lucas EA (1999). Buoyancy and diving behavior in mosquito pupae.. Journal of the American Mosquito Control Association.

[37] Romoser WS, Lerdthusnee K, Berry RL, Kittayapong P (1994). Effect of mechanical shock on hydrostatic balance and survival of mosquito pupae.. Journal of the American Mosquito Control Association.

[38] Martens WJM, Jetten TH, Rotmans J, Niessen LW (1995). Climate change and vector-borne diseases- a global modelling perspective.. Global Environmental Change.

[39] Lines J, Harrington R, Stork NE (1995). The effects of climatic and land-use changes on the insect vectors of human disease.. Insects in a changing environment.

[40] Shaman J, Day JF (2007). Reproductive phase locking of mosquito populations in response to rainfall frequency.. PLoS ONE.

